# Food shortages, stockpiling and panic buying ahead of Brexit as reported by the British media: a mixed methods content analysis

**DOI:** 10.1186/s12889-022-12548-8

**Published:** 2022-01-31

**Authors:** Paul C. Coleman, Fatima Dhaif, Oyinlola Oyebode

**Affiliations:** Warwick Medical School, The University of Warwick, Coventry, CV4 7AL UK

**Keywords:** Panic buying, Food shortages, Brexit, Media analysis

## Abstract

**Background:**

On 23 June 2016, the United Kingdom voted to leave the European Union. From that date until the UK left the EU in January 2021, there were frequent warnings from industry and government sources of potential disruption to the food supply chain and possible food shortages. Over this period, the media had an important role in communicating on the potential impacts of Brexit. This study examines how food supply and demand, in the context of Brexit, was portrayed by the British media.

**Methods:**

The study consisted of two components: (1) a quantitative analysis measuring frequency of reporting and information sources for articles on food supply and demand in the context of Brexit, in three daily newspapers, between January 2015 and January 2020; and (2) a content analysis exploring key themes and media framing of relevant issues in a subset of articles.

**Results:**

Reports by the media about the impact of Brexit on the UK food system were largely absent in the six months before the UK voted to leave the EU in June 2016, increasing in frequency from mid-2018 onward, peaking in mid-2019 following the appointment of Boris Johnson as prime minister. Five themes were developed from included articles: food shortages/panic buying (appearing in 96% of articles); food chain disruption (86%); economic impacts (80%); preparation and stockpiling by the government/food sector (63%) and preparation and stockpiling by individuals (22%).

**Conclusion:**

Government messaging sought to reassure the public that even under a worst-case scenario there would be no food shortages. These messages, however, contradicted warnings in the media of disruption to the food supply chain and food shortages. The media further reinforced this narrative of potential food shortages by reporting on the experiences of those preparing for Brexit by stockpiling food. The media must consider the impact of their messaging on public behaviour, as even imagined food shortages can instigate stockpiling and panic buying behaviour, as observed during the COVID-19 pandemic.

## Background

On 23 June 2016, the United Kingdom (UK) voted to leave the European Union (EU), an event known as ‘Brexit’. Prior to the UK leaving the EU there were warnings of potential disruption to the food supply chain and food shortages from the House of Commons [1], academics [2–4], leaked government documents [5] and industry experts [6].

For the general population, an important source of information on food supply chain disruption and food shortages was the mass media [7]. The nature of information conveyed through the media, including what gets reported, can have a powerful effect on public knowledge, attitudes and behaviours [8–10]. The mass media often oversimplified Brexit, avoiding the complexity of debates on topics such as the economy, immigration and trade [11–13]. Previous research exploring how the media reported on food, farming and agriculture found media outlets to select and promote issues that reinforced their own political agenda [7], a process known as ‘agenda setting’ [14]. This research, however, only focused on a three-month period in 2018 and did not explore how the media communicated risks associated with supply chain disruption and food shortages.

While there is no evidence from Kantar import data to suggest there were any food shortages experienced by consumers immediately following the UK leaving the EU [15–17], there was an 83% decline in fish and shellfish exports to the EU in early 2021 and substantial disruption to supply chains from mid to late 2021. It has been suggested that supply chain disruption, including a shortage of HGV drivers, petrol shortages in September 2021 and 17% of adults reporting food shortages in October 2021 [18], were a consequence of both the Covid-19 pandemic and impacts of Brexit [18, 19]. Consequently, understanding media reporting in the period leading up to the UK exiting the EU will provide a novel insight into how the risk of food supply chain disruption was communicated with the general public, including sources of information that were most frequently cited (e.g., government sources, food sector representatives or academics and experts) and whether messaging was balanced or disproportionate. This is of particular importance given the vulnerabilities in the UK food system highlighted during the Covid-19 pandemic and potential role of the media in instigating panic buying behaviour [20, 21]. However, it must be noted that media warnings of food supply chain disruption and food shortages were often associated with a no-deal scenario, an event that was not borne out.

The purpose of this research is to understand how issues of food shortages, stockpiling and panic buying, were portrayed by the British media between the build up to the EU referendum in 2016 and the UK officially leaving the EU single market in 2020. Specifically, this analysis will explore (i) trends and frequency of articles on the food system in relation to Brexit (ii) frequency with which these articles provide information from key sources (government, food sector, academics and experts) and (iii) key themes and framing in media reporting. A mixed methods design is used in which complementary methods allow an expansion of the breadth and range of enquiry.

## Methods

### Sampling

This study contained two components: (1) a quantitative analysis measuring trends in reporting of food chain disruption, and information sources, in three daily print and online British newspapers: the *Daily Mail*, *The Guardian* and the *Metro*; and (2) a content analysis exploring key themes and media framing of relevant issues in a subset of articles. Media outlets were selected based on levels of readership. The *Daily Mail* and the *Metro* being the two most widely read print newspapers and *The Guardian* the most read digital newspaper in the UK [22]. There are also notable differences in the demographic profile of readers. The *Daily Mail* attracts a majority of readers aged 65 and over in positions of semi-skilled labour; the *Metro* attracts a majority of readers aged 16 – 24 from a diverse range of ethnic groups; and *The Guardian* attracts primarily readers aged 16 – 24 in administrative/professional positions [22].

**Fig. 1:**
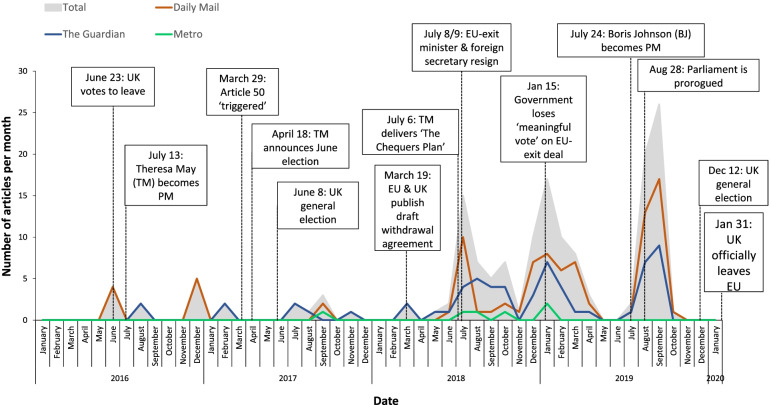
Systematic Review of Articles

Articles were accessed via the electronic archive of Nexis UK database (www.nexis.com) using the search term ‘Brexit’ AND (‘food shortage’ OR ‘food buying’ OR ‘food stockpiling’ OR ‘food availability OR ‘food supply’) between 01 January 2015 (6 months before the EU referendum) and 31 January 2020 (the day the UK officially left the EU). The original search identified 506 articles. Articles were screened by two researchers (PC and FD) and included if published in one of the three media outlets and if they contained two or more sentences focusing on food. Articles were excluded if they were a duplicate, letter or comment-piece (Fig. 1), 157 articles met the inclusion criteria.

### Component 1: Trends and frequency of articles on food and Brexit, and their key sources

A temporal analysis was conducted mapping coverage of Brexit and the food system in the three media outlets (*n* = 157). The timing of key events related to Brexit was demarcated against frequency of articles published. Frequency of articles including information attributed to different sources was tabulated.

### Component 2: Key themes and framing in media reporting

A content analysis was performed on a subset of 50 articles (due to time and resource constraints we were unable to review all 157 articles) using a combined deductive-inductive approach following the process outlined by Elo & Kyngäs [23]. All articles meeting inclusion criteria were stratified by publication date and media outlet and a random number generator used to select 50 articles.

During a preparation phase, articles were read and discussed by the research team and nascent codes identified. Articles were then reviewed by one researcher (PC) and codes further developed. Articles continued to be reviewed until three consecutive articles had been reviewed with no new codes developed. All articles were then coded according to the final set of codes by one researcher (PC). Codes were consolidated and grouped together to create a systematic framework, consisting of five themes. Codes and themes were discussed with the research team throughout. All coding was performed using NVivo 12.

## Results

### Component 1: Trends and frequency of articles on food and Brexit, and their key sources

**Fig. 2:**
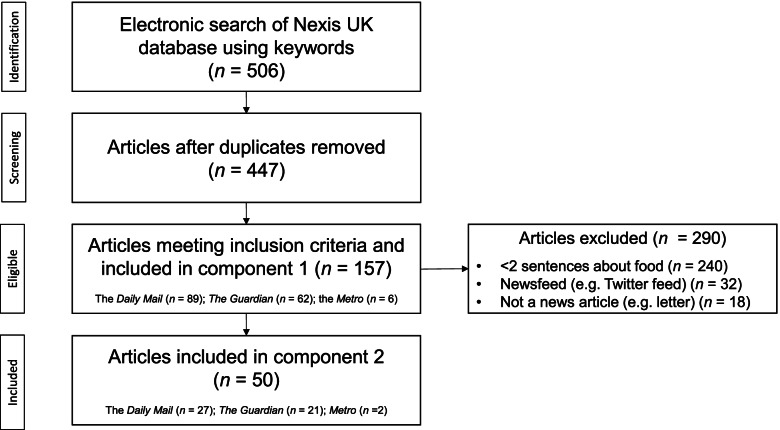
Total number of articles printed per month in the *Daily Mail*, *The Guardian* and the *Metro* which have at least two occurrences of the word ‘food’ in relation to Brexit and the timing of key events related to Brexit

In total, 157 articles were identified that contained at least two references to the word ‘food’ in relation to Brexit. Fig. 2 shows the highest number of articles in one month were published in September 2019 (*n* = 26; mean per month = 3), around the time the UK Parliament was ordered to be prorogued and the December general election being announced. Smaller peaks were observed in January 2019 (*n* = 17), at the time the House of Commons voted against the Government’s EU-withdrawal agreement and in July 2018 (*n* = 15).

Among the 50 articles reviewed in detail, approximately half of articles in the *Daily Mail* (56%) and *The Guardian* (48%) included information attributed to government sources. Only 7% of *Daily Mail* and 5% of *Guardian* articles included information attributed to opposition parties (Table 1). No information from government or opposition parties were identified in *Metro* articles.

**Table 1:** Frequency of articles including information attributed to different sources by media outlet

Media outlet	Academics & Experts % (*n*)	Food sector % (*n*)	Government % (*n*)	Opposition parties % (*n*)
*Daily Mail* (*n* = 27)	7 (2)	37 (10)	56 (15)	7 (2)
*The Guardian* (*n* = 21)	33 (7)	48 (10)	48 (10)	5 (1)
*Metro* (*n* = 2)	100 (2)	50 (1)	0 (0)	0 (0)

Component 2: Key themes and framing in media reporting.

Data from 50 articles reviewed in detail were coded into five themes (Table 2): ‘food shortages and panic buying’ (appearing in 96% of articles); ‘food supply chain disruption’ (86%); ‘economic impacts of Brexit’ (80%) ‘preparation and stockpiling by the government and food sector’ (63%) and ‘preparation and stockpiling by individuals’ (22%).

**Table 2:** Examples of data under the five themes in the systematic framework used to code the articles

Theme	Examples
Food shortages & panic buying (occurred in 96% of coded studies)	‘Though there would not be an overall shortage of food, the document says certain types of fresh food supply will decrease’ The *Daily Mail* 12 September 2019
	‘A No-deal outcome means there is a danger of empty shelves in shops. That could cause panic among consumers’ The *Daily Mail* 12 March 2019
	‘Councils around the country fear Brexit will lead to food shortages’ The *Metro* 2 August 2018
Food supply chain disruption (occurred in 86% of coded studies)	‘The government is holding talks with distributors after realising that the UK has a dire shortage of the “right sort” of pallets to import and export goods in the event of a no-deal Brexit’ *The Guardian* 26 February 2019
	‘The situation could be further worsened post-Brexit by a shortfall in labour, as workers who have enjoyed freedom of movement across the EU and come to Britain would no longer be available to pick home-grown fruit and vegetables’ The *Daily Mail* 26 June 2016
	‘There are fears a no-deal Brexit could leave supply chains disrupted because of new customs checks seizing up the Dover-Calais ferry route’ The *Daily Mail* 31 January 2019
Economic Impacts of Brexit (occurred in 80% of coded studies)	‘An average of 55% of farm income comes from the EU’s reviled common agriculture policy … losing these would cut swathes through agriculture and the landscape’ *The Guardian* 01 August 2017
	‘We already heard earlier this week from the food and drink industry that … the price of what’s left will rise’ The *Daily Mail* 31 January 2019
	‘more than a million workers employed in the food supply chain … consequences for working-class communities across the country will be devastating and long lasting’ *The Guardian* 20 August 2019
Preparation & Stockpiling: government and food sector (occurred in 63% of coded studies)	‘Brexit Secretary Dominic Raab insisted today the Government was not stockpiling food in case of a no-deal Brexit’ The Daily Mail 24 July 2018
	‘A leading ice cream maker revealed it was stockpiling Magnums and tubs of Ben and Jerry’s’ The *Daily Mail* 31 January 2019
	‘Schools have been advised to speak to suppliers to see if they can purchase food “off contract” and to ascertain whether they have stockpiles sufficient for an emergency’ *The Guardian* 21 March 2019
Preparation & Stockpiling: individuals (occurred in 22% of coded studies)	‘The most popular food items people wanted to store were staples such as flour, olive oil, dried pasta, rice, powdered milk, coffee and tinned items such as tomatoes and sardines’ *The Guardian* 09 August 2018
	‘Several readers said they were stockpiling store cupboard items such as tinned food and dried milk’ *The Guardian* 14 January 2019
	‘Wine, toilet roll and pasta are the most common items panicked shoppers are stockpiling in preparation for a no-deal Brexit, analysis of Mumsnet revealed’ The *Daily Mail* 8 April 2019

### Theme 1: Food shortages and panic buying

Themes of food shortages and panic buying were identified in most articles. Reports of the two were often inter-related, with panic buying positioned as both a cause and consequence of food shortages.

There were mixed reports on whether the government was anticipating food shortages. Quoting the Operation Yellowhammer report, the *Daily Mail* and *The Guardian* reported the government does not anticipate ‘*an overall shortage of food*’ (11 September 2019). In the same article, the *Daily Mail* reported of a ‘*risk that panic buying will cause or exacerbate food supply disruption*’ leading to ‘*reduced availability and choice’* of food items.

The food sector frequently warned of food shortages, particularly of perishable goods (e.g., The *Metro* 29 January 2019), with the CEO of Sainsbury’s warning that food will be left ‘*rotting at borders*’ (The *Daily Mail* 28 November 2018). The food sector also claimed that food shortages would result in reduced quality and standards (*The Guardian* 25 October 2018), with the Food Research Collaboration advising of ‘*Government … plans … to suspend food controls if there are any delays to imports of perishable foods at our borders*’ (The *Daily Mail* 24 July 2018).

### Theme 2: Food supply chain disruption

All three media outlets reported on possible disruption to the supply of food entering the UK. The food sector appeared to be most vocal in expressing concern, with Co-op Food and Marks and Spencer warning that food imports, particularly perishable goods, ‘*could reduce by 87 per cent after a no-deal*’ due to new customs checks (The *Metro* 29 June 2019). Articles also included warnings from the food sector of the impact of reduced migrant labour, with food producers warning that ‘*British fruit and vegetables would all but vanish from shops if … foreign workers … are no longer able to come to the UK*’ (*The Guardian* 16 August 2016).

The government expressed concern of disruption to the food supply chain. Media outlets, again citing the Operation Yellowhammer report, warned of ‘*significant and prolonged*’ disruption lasting three to six months (The *Daily Mail* 11 and 12 September).

### Theme 3: Economic impacts of Brexit on the food system

Reports on the economic impact of Brexit focused primarily on the consequences of a no-deal Brexit. There were warnings from the government, food sector and academics that a no-deal Brexit could result in increased food prices, with researchers from Imperial College London warning that ‘*rising fruit and veg* [etable] *prices after a no-deal could lead to thousands more deaths from heart attacks and strokes*’ (The *Metro* 29 January 2019). Two articles in *The Guardian* reported on the impact of rising food costs on exacerbating existing health inequalities, specifically the impact on ‘*children’s health, safety and education*” (*The Guardian* 18 September 2019).

### Theme 4: Preparation and stockpiling by the government and food sector

Media outlets presented mixed messages on whether the government and food sector were stockpiling food. The government was portrayed as advising the food sector – but not individuals – on how to prepare for Brexit (*The Daily Mail* 25 July 2018), while the food sector was portrayed as unprepared and uncooperative (The *Daily Mail* 28 November 2018). The Food Chain Emergency Liaison Group (FLEG) was accurately presented as the government team responsible for protecting the UK’s food system from the impacts of a no-deal Brexit. The *Daily Mail* described FLEG as a ‘*120-strong food supply team made up of senior Whitehall officials*’ (12 March 2019).

The food sector was presented by the media, particularly the *Daily Mail*, as not being prepared for Brexit and described as ‘*in the dark*’ (The *Daily Mail* 28 November 2018). Articles also referred to ‘*friction between the Government and supermarkets*’ due to a lack of preparation by the food sector (The *Daily Mail* 12 March 2019).

Several articles reported that stockpiling by the food sector was either underway (*The Daily Mail* 25 July 2018) or would be in the near future (*The Daily Mail* 28 November 2018). Others articles reported that the ability of the food sector to stockpile food was ‘*very limited*’ (*The Daily Mail* 28 November 2018) due to difficulties in storing perishable food and a lack of storage space (*The Daily Mail* 28 November 2018; *The Guardian* 26 July 2018).

### Theme 5: Preparation and stockpiling by individuals

At the individual level, there was a fear of food shortages and panic buying. One individual described panic buying as ‘*the most likely outcome*’ of Brexit (*The Guardian* 14 January 2019). There was often a distinction between ‘normal’ people preparing for Brexit (seen as a positive behaviour) and ‘stockpilers’ (seen as an extreme behaviour). Individuals preparing for Brexit wanted to distance themselves from ‘stockpilers’ who were portrayed as alarmist and ‘*barmy*’ (The *Daily Mail* 02 December 2019) due to undertaking extreme behaviour such as creating ‘*a Brexit bunker in the cellar*’ (The *Daily Mail* 22 January 2019). There were, however, similarities in the reported opinions of both groups, particularly a lack of trust in the government to protect them ‘*in the event of a crisis*’ (*The Guardian* 9 August 2018).

Individuals reported stockpiling non-perishable items such as flour and pasta (The *Daily Mail* 08 April 2019), ‘*luxury’* items such as chocolate (*The Guardian* 9 August 2018), as well as purchasing polytunnels, shelving racks and fridge freezers (The *Daily Mail* 2 December 2018, *The Guardian* 14 January 2019). People were quoted as hoping to stockpile between one to three months’ supply of food and said they would donate excess food to food banks/charities if food supplies were not affected (*The Guardian* 09 August 2018). The media recognised their role in influencing human behaviour, with *The Guardian* reporting that ‘*newspaper headlines [may] become self-fulfilling very quickly: I foresee panic buying’*’ (*The Guardian* 14 January 2019).

## Discussion

This study aimed to explore how issues of food shortages, stockpiling and panic buying, were portrayed by the British media between the build up to the EU referendum in 2016 and the UK officially leaving the EU single market in 2020. Media reports on the impact of Brexit on the UK food system were largely absent in the six months before the UK voted to leave the EU in June 2016, only increasing in frequency from mid-2018. The three media outlets included in this study cited information from government sources more frequently than information from the food sector, academics and other experts or opposition party politicians. The key themes developed during the analysis were food shortages and panic buying; food supply chain disruption; economic impacts of Brexit and preparation and stockpiling by the government, food sector and individuals.

### Trends and frequency of articles on food and Brexit

Reports by the media about the impact of Brexit on the UK food system were largely absent in the six months before the UK voted to leave the EU in June 2016 and only increased in frequency from mid-2018 onward, peaking in mid-2019 following the appointment of Boris Johnson as prime minister. The relatively late emergence of articles mentioning ‘food’ suggests that the impacts of Brexit on the food system were not frequent themes of Brexit discourse during the Brexit campaign or during the early stages of UK-EU trade negotiations. Indeed, causal mapping of televised debates during the build up to the UK-EU referendum found the most dominant themes to be immigration, impacts on the economy, job opportunities and ‘taking back control’, with no reference to the food or farming sector [24].

There were three main peaks in media reports on the impacts of Brexit on the food system, coinciding with Theresa May delivering the Brexit whitepaper (The ‘Chequers Plan’) in July 2018; the government losing the ‘meaningful vote’ on their EU-exit deal in January 2019 and the lead up to the UK submitting a new EU-exit plan to the EU in October 2019. These events all coincided with the government outlining plans for the future of the UK food sector, including farming subsidies to replace the EU’s Common Agricultural Policy and impacts of tariff and non-tariff measures (e.g., sanitary and phytosanitary measures) on food imports and exports [25]. Indeed, the disproportionate volume of EU legislation governing the food and farming sector [6] made the process of withdrawing from EU institutions highly complex and contributed towards delays to the UK’s departure from the EU and numerous rejections by Parliament of the government’s Withdrawal Agreements [26].

### Key sources from which stories emanated

Media articles cited information from government sources (50% of articles) more frequently than information attributed to food sector representatives (42% of articles), academics and other experts (22%) or opposition party politicians (6% of articles). While previous studies have criticised the media’s disproportionate representation of politicians initially involved in shaping the Brexit campaign [26, 27], it is well recognised that politicians use the media as a tool for communicating with the public and a way to influence public opinion [28], and these findings support previous research which also found political voices to feature more prominently than other stakeholder groups [7].

Representation of government figures, food sector representatives and opposition parties were broadly similar across media outlets, however, academics and food system experts featured more frequently in The Guardian (33% of articles) than the Daily Mail (7% of articles). In line with previous research, which has shown media outlets to select and promote issues that reinforce their own political agenda [7], it is plausible that concerns voiced by academics of food shortages and disruption to the food supply were more aligned with the anti-Brexit position of The Guardian than the pro-Brexit narrative of the Daily Mail, resulting in increased prominence and reporting by The Guardian.

### Themes and framing of relevant issues

The key themes developed during the analysis were food shortages and panic buying; food supply chain disruption; economic impacts of Brexit and preparation and stockpiling by the government, food sector and individuals. The theme of ‘food shortages and panic buying’ occurred in 96% of coded articles. Government communication around potential food shortages was framed as being chaotic and confusing [29]. For example, articles often included both reassuring quotes from the government that there was no risk of food shortages, followed by contradictory warnings of future panic buying and food supply chain disruption from government reports (e.g., Operation Yellowhammer). While it is not possible to elucidate what impact this mixed messaging had on human behaviour, evidence from both the COVID-19 pandemic and 2007/08 food crisis have shown it is often the act of panic buying itself, rather than disruption to the food supply, that results in product shortages at the consumer-level [30, 31]. As media messaging can promote either reassurance or anxiety on the issue of food shortages and cause anything from no change in behaviour, to better preparation for real challenges or panic buying in response to real or imagined challenges, the media must consider what impact their messages may have on human behaviour.

The theme of food supply chain disruption, which occurred in 86% of articles, also had parallels with government communication during the COVID-19 pandemic with an emphasis on reassuring the public there will be no disruption to the food supply chain [32]. These messages, however, contradicted warnings in the media, often within the same article, of disruption to the food supply chain and reduced availability of food items. Warnings of food supply chain disruption were often associated with a no-deal scenario, an event that was not borne out, with most articles coinciding with the government losing the ‘meaningful vote’ in January 2019 and parliament being prorogued in September 2019. Findings reported here agree with previous evidence tracking the emergence of the no-deal scenario (using reports from parliamentary debates and UK internet searches) emerging at a late stage in the Brexit process amid growing disillusionment among Brexit supports [26].

Reports on the economic impacts of Brexit occurred in 80% of articles and typically focused on a no-deal scenario resulting in increased food prices as a result of higher import tariffs. A further socio-economic dimension, reported by two Guardian articles but not the Daily Mail, was that disruption to the food supply chain would disproportionately impact those already struggling to access food. A similar scenario was observed during the COVID-19 pandemic when disruption to the food supply chain and economic instability contributed towards the number of individuals experiencing food insecurity increasing by 250% over pre-Covid-19 levels, to 2.9 million people [33].

Reports on preparation and stockpiling by the government and individuals occurred in 63% and 22% of articles, respectively. It is possible that the media were using lived experiences of their readers to highlight the importance of stockpiling food as a way to prepare for Brexit. This phenomenon, known as ‘media persuading’, has the potential to influence behaviour at the population level [34]. For example, an increase in the volume of media reporting on transmission of the Zika virus within the United States was associated with an increase in the perception of risk in contracting the virus and protective behaviours, such as consulting medical professionals [35], while an experimental study in Taiwan found media reporting on swine flu to invoke a sense of vulnerability rather than instigating protective behaviours, such as preparing for the outbreak [36]. In relation to Brexit, there was even recognition by *The Guardian* newspaper that newspaper headlines could become self-fulfilling, as well as requests for readers to contact media outlets to report on their own stockpiling stories. These findings are particularly timely given the potential role of the media in instigating stockpiling behaviour during the COVID-19 pandemic by focusing on images of empty food shelves, rather than centring the message that there was no overall shortage of food in the supply chain [20, 31, 37, 38].

### Implications

There is an opportunity for the Editors’ Code of Practice [39], enforced in the UK by the Independent Press Standards Organisation, to consider what impact sensationalised reporting has on the health and wellbeing of readers. While the Editors’ Code of Practice considers factors such as taking ‘*care not to publish inaccurate, misleading or distorted information*’, there is currently no requirement to consider what behavioural response may be induced, as a result of sensationalised media stories. In part, this sensationalised reporting is a result of oversimplifying complex arguments, as observed in media portrayals of immigration throughout the Brexit campaign [13]. This tension between delivering accurate reporting and a need to attract readers has previously been shown to negatively impact health and wellbeing [40] with a wide body of research showing that media reports on suicides and body size can influence suicidal behaviour and eating disorders, respectively, particularly among vulnerable groups and young people [41–44].

### Limitations

There are limitations associated with this study. By restricting this study to three media sources there is a focus on information targeting a subset of the general population, however the three media outlets were selected based on their appeal to different demographics and include the two most widely read print newspapers and the most widely read digital newspaper in the UK. Component 2 of this study included a subset of 50 articles due to time and resource constraints, further themes may have been developed if additional articles had been included, however no new themes were developed after screening 30 articles, so it is possible that data saturation had been achieved. While findings do not seek to prove causal effects of a relationship between media reporting and panic buying, they do provide innovative insights into how the media report on potential disruption to the food system. This study did not consider reporting of food shortages and panic buying by social medial, an important area of future study given the potential role of social media platforms in shaping fear and consumer responses during the COVID-19 pandemic [45]. Finally, it is to be expected that the search terms utilised in this study (e.g., ‘food shortage’) would identify media reports that emphasised potential threats to UK food security rather than reports that Brexit would not impact the UK food system. However, we also included more neutral terms (food supply, food availability, food buying). Nevertheless, the articles identified did not simply report concerns raised by the food sector and other stakeholders, there was a focus on individual stockpilers and ‘preppers’, which seemed to normalise irrational stockpiling behaviour.

## Conclusion

Media messaging on potential food shortages and panic buying was often unclear and inconsistent. Media communication, however, seemed to mirror chaotic government communication, which both sought to reassure the public that there would be no food shortages, while simultaneously warning of food supply chain disruption and food shortages. The media then reinforced this narrative of potential food shortages by reporting on the experiences of those preparing for Brexit by stockpiling food. The media must consider the impact of their messaging on public behaviour, as even imagined food shortages can instigate stockpiling and panic buying behaviour, as observed during the COVID-19 pandemic.

## Data Availability

The datasets used and/or analysed during the current study are available from the corresponding author on reasonable request.

## Abbreviations

EU: European Union
UK: United Kingdom
